# Local capacity for policy development and implementation

**DOI:** 10.2471/BLT.25.294222

**Published:** 2025-07-01

**Authors:** Aklilu Azazh, Jacinta Nzinga, Joseph Ngonzi, Kidist Bartolomeos

**Affiliations:** a College of Health Sciences, Department of Emergency Medicine, Addis Abeba University, P.O. box 9086, Addis Abeba, Ethiopia.; b Health Systems and Research Ethics Department, KEMRI-Wellcome Trust Research Programme, Nairobi, Kenya.; c Mbarara University of Science and Technology, Mbarara, Uganda.; d Science Division, World Health Organization, Geneva, Switzerland.

The World Health Organization (WHO) plays a core normative and technical role in providing global reference standards, policy options and guidelines, and other normative products. This function has been reinforced in the Fourteenth Global Programme of Work,^1^ which highlights WHO’s responsibility for developing, implementing and monitoring and evaluating global guidance. This role supports and enables the work of Member States and partners at all levels of the health system.^1^ Timely and effective uptake of evidence-based guidance into policy and practice is critical to build efficient and resilient health systems and achieve better health outcomes.^2^ However, guidance will only produce positive health outcomes if critical national structures, policies, decision-makers and resources are available to implement and monitor progress. To be impactful, WHO normative products should be locally contextualized and relevant to countries. Such contextualization cannot be achieved without understanding the lived experience of the people implementing the normative products, the contexts in which they work, and the supports and challenges they encounter in the process of change. By understanding how end-users will use recommendations, WHO can develop normative products in a way that responds to diverse constraints and unique cultural contexts. Here we provide a reflection on common underlying challenges in improving guideline adaptation and uptake across four African countries (Ethiopia, Kenya, Uganda and United Republic of Tanzania), and identify where investment is required. 

WHO guidelines are grounded in scientific evidence and often serve as the basis of many national health policies. With technological progress, WHO-issued guidelines are transitioning from traditional paper-based formats to technology-enabled, living documents that can be immediately updated, making guidance development a dynamic process.^3^ This approach offers regulators and policy-makers the latest scientific information in real-time, as demonstrated during the coronavirus disease 2019 pandemic.^4^ The impact of these innovations is maximized when researchers, policy-makers and implementors are engaged early in the process.^5^ Innovations also require governments to have the capacity to accurately interpret the guideline recommendations and, when necessary, adapt them to the local circumstances. Lack of human, financial and infrastructure resources for evidence-based adaptation and implementation is one of the systemic challenges that may arise.^5^ While vertical implementation programmes might achieve results, they are often limited to a geographic area, have an end date, and are often externally funded. Sustained investment in governance, leadership and coordination structures that allow adaptation is needed to achieve impact at the national level. 

In the WHO European Region, guideline adaptation programmes are assisting governments’ technical departments in building capacity in contextualization and institutionalizing adaptation efforts.^6^ Similarly, in the WHO Eastern Mediterranean Region, WHO is supporting governments to develop national strategies based on a regional action plan.^7^ In the WHO African Region, since 2021, WHO has supported governments in conducting rapid research on guideline adaptation and uptake across five countries (Cabo Verde, Ethiopia, Kenya, Uganda and United Republic of Tanzania).^5^ The experience from these three WHO regions shows the practical steps needed to efficiently implement and contextualize WHO guidelines, and underscores the importance of investing in the necessary resources.

Researchers supporting guideline development and systematic adaptation of published guidelines have analysed the system-level and capacity-related challenges that hinder evidence-based adaptation in low-resource settings.^9^ Findings include that, for sustained action and change, institutionalizing capacity development and related skills in guideline development is needed – in addition to the required governance and coordination structures. As well, investment in human capacity (for those involved in guidance development) and infrastructure is needed to contextualize global guidance and its dissemination in ways that are effective and timely. Products should be available in formats (including decision aids, checklists) that are accessible to users and can be easily integrated into locally developed digital products. Countries also need to adopt an evidence-based and data-driven approach when prioritizing global recommendations in national investment decisions. 

In line with these findings, the World Health Assembly resolution WHA78 *Strengthening national capacities in evidence-based decision-making for the uptake and impact of norms and standards*^10^ calls for action to address the persistent gaps in the uptake of global norms and standards and barriers to achieving equitable health outcomes in countries.
